# Intrastructural Help: Harnessing T Helper Cells Induced by Licensed Vaccines for Improvement of HIV Env Antibody Responses to Virus-Like Particle Vaccines

**DOI:** 10.1128/JVI.00141-18

**Published:** 2018-06-29

**Authors:** Hassan Elsayed, Ghulam Nabi, William J. McKinstry, Keith K. Khoo, Johnson Mak, Andres M. Salazar, Matthias Tenbusch, Vladimir Temchura, Klaus Überla

**Affiliations:** a Institute of Clinical and Molecular Virology, University Hospital Erlangen, Friedrich-Alexander Universität Erlangen-Nürnberg, Erlangen, Germany; b Department of Microbial Biotechnology, Genetic Engineering and Biotechnology Division, National Research Centre, Dokki, Giza, Egypt; c Department of Molecular and Medical Virology, Ruhr-University Bochum, Bochum, Germany; d CSIRO Manufacturing, Parkville, Victoria, Australia; e School of Medicine, Deakin University, Geelong, Australia; f Institute for Glycomics, Griffith University, Gold Coast, Queensland, Australia; g Oncovir, Inc., Washington, DC, USA; Ulm University Medical Center

**Keywords:** intrastructural help, T helper cells, HIV, Env, antibody, vaccine, VLP, IgG subtype

## Abstract

Induction of persistent antibody responses by vaccination is generally thought to depend on efficient help by T follicular helper cells. Since the T helper cell response to HIV Env may not be optimal, we explored the possibility of improving the HIV Env antibody response to virus-like particle (VLP) vaccines by recruiting T helper cells induced by commonly used licensed vaccines to provide help for Env-specific B cells. B cells specific for the surface protein of a VLP can internalize the entire VLP and thus present peptides derived from the surface and core proteins on their major histocompatibility complex class II (MHC-II) molecules. This allows T helper cells specific for the core protein to provide intrastructural help for B cells recognizing the surface protein. Consistently, priming mice with an adjuvanted Gag protein vaccine enhanced the HIV Env antibody response to subsequent booster immunizations with HIV VLPs. To harness T helper cells induced by the licensed Tetanolpur vaccines, HIV VLPs that contained T helper cell epitopes of tetanus toxoid were generated. Tetanol-immunized mice raised stronger antibody responses to immunizations with VLPs containing tetanus toxoid T helper cell epitopes but not to VLPs lacking these epitopes. Depending on the priming immunization, the IgG subtype response to HIV Env after the VLP immunization could also be modified. Thus, harnessing T helper cells induced by other vaccines appears to be a promising approach to improve the HIV Env antibody response to VLP vaccines.

**IMPORTANCE** Induction of HIV Env antibodies at sufficient levels with optimal Fc effector functions for durable protection remains a challenge. Efficient T cell help may be essential to induce such a desirable antibody response. Here, we provide proof of concept that T helper cells induced by a licensed vaccine can be harnessed to provide help for HIV Env-specific B cells and to modulate the Env-specific IgG subtype response.

## INTRODUCTION

**FIG 1:**
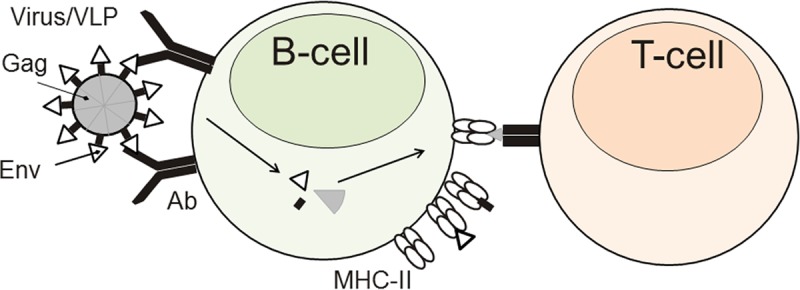
Mechanism of intrastructural help. Env-specific B cells encountering HIV virions or VLPs take up the entire particle in a BCR-dependent manner and present Env- and Gag-derived T helper cell epitopes on their MHC-II molecules. Therefore, Gag-specific T helper cells can provide help to Env-specific B cells.

Virus-like particle (VLP) vaccines have been shown to be highly immunogenic and to provide protection from a number of viral diseases (reviewed in reference 1). Due to the repetitive nature of their surface proteins, VLPs can trigger cross-linking of B cell receptors (BCRs) specific for the surface protein, which may partially explain the good antibody (Ab) responses observed after VLP immunization (2). Development of VLP vaccines against HIV faces a number of challenges. The diversity of circulating HIV strains requires Env immunogens that induce broadly reactive antibody responses to the native Env spikes on the virion. Therefore, Env immunogen design is a major focus of current HIV vaccine development efforts (reviewed in reference 3). In addition, induction of HIV Env antibodies at levels high enough to provide durable protection from HIV acquisition remains a challenge (4). Providing adequate T cell help may be a critical step for the development of vaccines inducing long-lasting antibody responses protecting from HIV acquisition. In mice, we observed that induction of primary anti-Env antibody responses by VLP immunization depends on T helper cells (5). However, T helper cell responses to HIV Env are generally weak during HIV infection (6), and HIV Env antibody responses in human vaccinees seem to rapidly decline (7). We previously showed that Gag-specific T helper cells induced by gene-based vaccines can provide help for HIV Env-specific B cells after booster immunizations with VLPs (8, 9). This intrastructural help (10–13) can be explained by uptake of entire VLPs by Env-specific B cells and subsequent presentation of Gag- and Env-derived T helper cell epitopes on major histocompatibility complex class II (MHC-II) molecules of the Env-specific B cells (Fig. 1). We now extend these observations to adjuvanted protein vaccines and provide proof of concept that T helper cells induced by a commonly used licensed protein vaccine can also be harnessed to deliver help for Env antibody responses. Thus, recruitment of preexisting T helper cells induced by licensed vaccines seems to be a promising novel strategy to optimize antibody responses to surface proteins of VLP vaccines.

## RESULTS

### Modulation of the HIV Env-specific antibody response by an adjuvanted HIV Gag protein immunization.

We previously observed that T helper cells induced by DNA or adenoviral vector vaccines encoding Gag or GagPol provided intrastructural help for Env antibody responses after VLP booster immunizations in mice (8, 9). To explore whether an adjuvanted protein vaccine could similarly modulate the HIV Env antibody response by intrastructural help, mice were first immunized with recombinant Gag protein adjuvanted with poly(ICLC), a Toll-like receptor 3 (TLR3) and MDA-5 ligand (14). Poly(ICLC) has been shown to be a strong inducer of T helper cell responses in mice (15) and nonhuman primates (16). Several reports (17, 18) also demonstrated that poly(ICLC) induces strong CD4^+^ T cell responses but weak CD8^+^ T cell responses.

**FIG 2:**
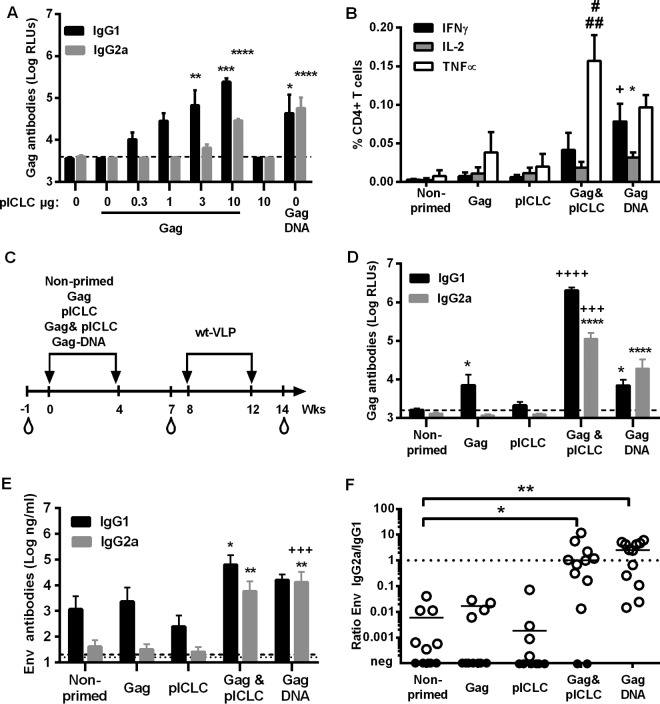
Intrastructural help by Gag immunogens. (A) BALB/c mice (*n* = 4 per group) were immunized once with 1 μg Gag and the indicated dose (μg) of poly(ICLC) (pICLC) or with 25 μg Gag DNA vaccine by i.m. DNA electroporation. Three weeks later, the antibody responses against Gag were analyzed at 1:500 serum dilutions. Shown are the mean values with the SEM for logarithmically transformed values for Gag IgG1 and IgG2a. *, *P* < 0.05; **, *P* < 0.01; ****, *P* < 0.001 (vaccine groups versus nonprimed; one-way ANOVA with Tukey's posttest). (B) Gag-specific CD4^+^ T cell responses were analyzed by intracellular cytokine staining for the indicated cytokines 2 weeks after a single i.m. injection of 1 μg Gag, 10 μg poly(ICLC) (pICLC), or the combination of Gag and pICLC or a single i.m. electroporation of 25 μg of a Gag DNA vaccine into BALB/c mice. Shown are the mean values with SEM for four animals per group (^+^, *P* < 0.05 versus PBS, Gag, and pICLC for IFN-γ; *, *P* < 0.05 versus PBS for IL-2; ##, *P* < 0.001 versus PBS and pICLC for TNF-α; #, *P* < 0.05 versus Gag for TNF-α [one-way ANOVA with Tukey's posttest]). (C) BALB/c mice (*n* = 11 or 12 per group) were immunized at weeks 0 and 4 with 1 μg Gag or 10 μg pICLC alone or in combination or with the Gag DNA vaccine. All primed and nonprimed mice were boosted at weeks 8 and 12 with the same VLP preparation containing Env and Gag, and naive sera were taken 1 week before the first immunization. (D) Antibody responses to Gag at 3 weeks after the second priming immunization at a serum dilution of 1:1,000. Shown are the mean values with SEM for 11 or 12 animals from two independent experiments. The dashed line represents the background of naive sera for Gag antibodies. For IgG1, *, *P* < 0.05 versus nonprimed; ^++++^, *P* < 0.001 versus nonprimed, Gag, pICLC, and Gag DNA (one-way ANOVA with Tukey's posttest). For IgG2a, ****, *P* < 0.0001 versus nonprimed, Gag, and pICLC; ^+++^, *P* < 0.001 versus Gag DNA (one-way ANOVA with Tukey's posttest). (E) Env-specific antibody responses 2 weeks after the second VLP booster immunization. Shown are the mean values with SEM for logarithmically transformed HIV Env antibody concentrations in 11 or 12 animals from two independent experiments. For IgG1, *, *P* < 0.05 versus pICLC (Kruskal-Wallis test with Dunn's posttest). For IgG2a, **, *P* < 0.01 versus nonprimed, Gag, and pICLC; ^+++^, *P* < 0.001 versus Gag and pICLC (Kruskal-Wallis test with Dunn's posttest). The dashed lower and upper lines represent the detection limits of the HIV Env-specific IgG2a and IgG1 antibody levels, respectively. (F) Env-specific IgG2a/IgG1 ratios 2 weeks after the second VLP immunization. The bars represent the median of the ratios for all animals of each group that were positive for both Env-specific IgG1 and IgG2a antibodies (open symbols). Samples that were under the limit of detection for IgG2a and/or IgG1 are shown by closed symbols. *, *P* < 0.05; **, *P* < 0.01 (Kruskal-Wallis test with Dunn's posttest; vaccine groups versus nonprimed).

The Gag antibody response after a single immunization with the adjuvanted Gag protein vaccine was compared to that after immunization with an HIV-Gag DNA vaccine to define a suitable concentration of the adjuvant. Mice were primed with a constant amount of Gag protein in the presence of increasing concentrations of poly(ICLC). Three weeks after the immunization, Gag-specific antibody levels directly correlated with the dose of the adjuvant. The minimal dose of poly(ICLC) required for significant enhancement of Gag-specific IgG1 and IgG2a antibody levels was 10 μg (Fig. 2A). We therefore selected this dose for all subsequent experiments. The adjuvanted Gag protein and the Gag DNA vaccines also induced Gag-specific T helper cells producing Th1 cytokines (Fig. 2B). Interestingly, the cytokine profiles differed between the two groups. The response in the adjuvanted Gag group was dominated by tumor necrosis factor alpha (TNF-α), while a more balanced gamma interferon (IFN-γ) and TNF-α production was observed in the Gag DNA group (Fig. 2B).

To explore the extent to which Gag-specific T helper cells provide intrastructural help, mice received two priming immunizations with different Gag immunogens or controls prior to two booster immunizations with the same VLPs (Fig. 2C). After two priming immunizations with the adjuvanted Gag protein vaccine, strong Gag-specific IgG1 and IgG2a antibody responses that exceeded the ones induced by the Gag DNA vaccine were observed (Fig. 2D). Nonadjuvanted Gag induced only a weak Gag-specific IgG1 response. Looking at the HIV Env antibody responses after the two VLP booster immunization, we noted that priming with adjuvanted Gag significantly enhanced HIV Env-specific IgG1 and IgG2a antibody levels, while Gag DNA priming enhanced only HIV Env-specific IgG2a antibody responses (Fig. 2E).

Since Fc effector functions may depend more on the ratio of IgG subtypes than on their absolute values, we also determined the ratio of HIV-1 Env-specific (IgG2a/IgG1) antibodies. Mice that were primed by either HIV Gag DNA or adjuvanted HIV Gag protein immunization had a significantly enhanced ratio of HIV-1 Env-specific (IgG2a/IgG1) antibodies (Fig. 2F), confirming that intrastructural help can modulate IgG subtype ratios.

### Generation of VLPs containing FrC of TT.

**FIG 3:**
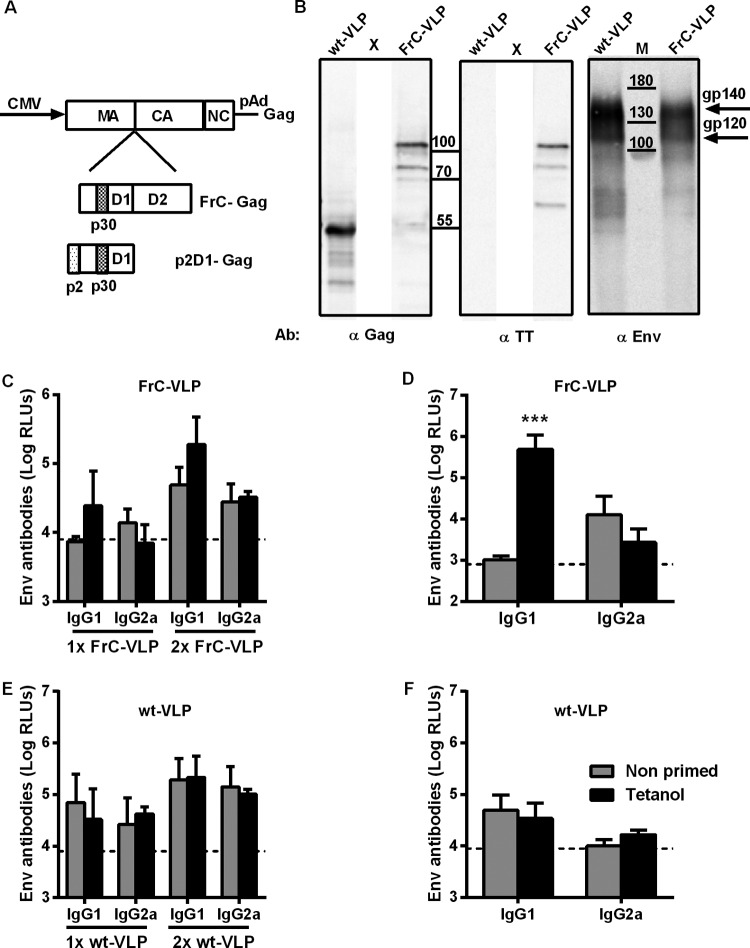
Intrastructural help by tetanus toxoid-specific T helper cells. (A) Map of Gag expression plasmids encoding wild-type Gag, an FrC-Gag fusion protein, or an p2D1-Gag fusion protein. (B) Western blot analyses of wild-type VLPs (wt-VLP) and FrC-VLPs with antibodies detecting the indicated proteins. X, positions of lanes removed from the Western blots. (C to F) Env-specific antibody responses in BALB/c mice primed twice with Tetanol and boosted once or twice with FrC-VLPs (C), BL/6 mice primed twice with Tetanol and boosted once with FrC-VLPs (D), BALB/c mice primed twice with Tetanol and boosted once or twice with wt-VLPs (E), and BL/6 mice primed twice with Tetanol and boosted once with wt-VLPs (F). The dashed lines represent the background observed in naive animals. Shown are the means and the SEM of logarithmically transformed RLU for 4 animals per group at a 1:250 serum dilution (C and E) and for 6 animals per group at a 1:100 serum dilution (D and F). ***, *P* < 0.001 (unpaired *t* test).

Having shown that an adjuvanted Gag protein vaccine can provide intrastructural help for HIV Env antibody responses triggered by a VLP vaccine, we asked whether T helper cells induced by a licensed protein vaccine, such as Tetanol, could be harnessed to provide intrastructural help for HIV VLPs. Since fragment C (FrC) of tetanus toxoid (TT) has been shown to efficiently harness T cell help for antibody responses in the context of fusion proteins (19), we inserted the coding sequence of fragment C of TT in frame between the genes for matrix (MA) and capsid (CA) of Gag (Fig. 3A). Insertions at this site have been shown previously to be compatible with particle formation and budding (33). Transient cotransfection of the FrC-Gag and Env expression plasmids into 293T cells led to the release of particles (FrC-VLP) that could be pelleted through a 35% sucrose cushion (Fig. 3B). Although the Env content of wild-type VLPs seemed to be higher, Env could also be detected readily in the FrC-VLPs (Fig. 3B). Western blot analysis with antibodies to Gag and fragment C of tetanus toxoid revealed that the FrC-Gag fusion protein migrated at a molecular weight of approximately 105 kDa, while the parental Gag had the expected molecular weight of 55 kDa (Fig. 3B).

### Influence of Tetanol immunization on the Env-specific IgG responses to VLP vaccines containing TT T helper cell epitopes.

To explore the effect of Tetanol immunization on the HIV Env antibody response to VLPs containing fragment C of TT, BALB/c mice were immunized twice with Tetanol (weeks 0 and 4) prior to one or two injections of VLPs containing fragment C. After the booster immunizations with VLPs containing fragment C, there was a trend to higher Env IgG1 antibody responses in Tetanol-primed mice receiving VLPs containing fragment C than in unprimed BALB/c mice, although this difference did not reach statistical significance (Fig. 3C). No increase in Env-specific IgG2a antibody responses was observed. Since BALB/c mice were reported to have a Th2 bias favoring IgG1 antibody responses, we also performed a Tetanol prime (weeks 0 and 4) and VLP boost (week 8) experiment in BL/6 mice. A strong increase in Env-specific IgG1 antibody levels was observed in BL/6 mice that had received prior Tetanol immunizations, while there was no effect on Env-specific IgG2a antibody levels (Fig. 3D). In both BALB/c and BL/6 mice, no Tetanol priming effect on Env IgG responses was observed after boosting with VLPs lacking fragment C of TT (Fig. 3E and F).

### Influence of TT-specific CD4^+^ T cells on the *in vivo* proliferative response of Ag-specific B cells to VLPs containing TT T helper cell epitopes.

**FIG 4:**
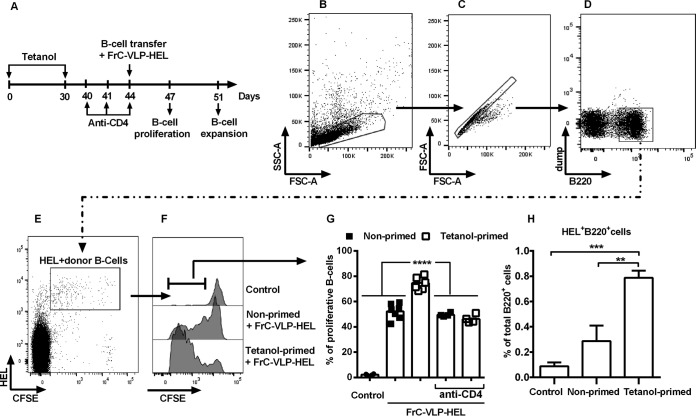
Enhancement of the proliferative response of naive antigen-specific B cells by intrastructural help. (A) BL/6 mice were primed s.c. twice over a 4-week interval with 10 μl of Tetanol per animal. Ten days after the last Tetanol injection, CD4 cells were depleted. Two weeks after the last Tetanol injection, naive B cells from SW-HEL mice were labeled with CFSE and adoptively transferred into nonprimed or Tetanol-primed mice with or without CD4 cell depletion. The acceptor animals were injected i.v. with FrC-VLPs containing membrane-anchored HEL (HEL-VLP). Three days later, splenocytes of these mice and of untreated controls were stained with Alexa 647-conjugated HEL and anti-B220–APC-eFluor780 antibody. (B to F) Gating strategy for the analysis of *in vivo* proliferating CFSE^+^ HEL^+^ B220^+^ B cells. (B) Gating on lymphoid cells according to their forward scatter and sideward scatter. (C) Elimination of doublets by FSC-A versus. FSC-H gating. (D) A dump channel was used to exclude autofluorescent cells among B220-positive cell singlets. (E) HEL- and CFSE-positive cells were selected. (F) Gating on proliferating CFSE^+^ HEL^+^ B220^+^ B cells. Representative data are shown. (G) The bars represent mean values of percent proliferating cells for each experimental group; values for individual mice are also shown. ***, *P* < 0.001 (one-way ANOVA with Tukey's posttest). (H) The bars represent expansion of HEL-specific B cells (percentage of HEL^+^ B220^+^ B cells among the total B220^+^ B cell population) in nonprimed and Tetanol-primed animals versus controls. **, *P* < 0.001; ***, *P* < 0.001 (one-way ANOVA with Tukey's posttest; vaccine groups versus control).

To corroborate our hypothesis that TT-specific CD4^+^ T cells provided direct help to HIV-1 Env-specific B cells, we used the hen egg lysozyme (HEL) model antigen (Ag) and established an *in vivo* proliferation assay for antigen-specific B cell responses that are dependent on TT-specific T helper cells (Fig. 4A to F). Injection of VLPs displaying HEL on their surface into mice that received carboxyfluorescein succinimidyl ester (CFSE)-labeled naive HEL-specific B cells 4 h prior to the VLP injection led to proliferation of the HEL-specific B cells (Fig. 4G). Priming mice with Tetanol prior to transfer of CFSE-labeled HEL-specific B cells and injection of VLPs containing HEL on their surface and fragment C inside enhanced the proliferative response of the HEL-specific B cells in comparison to nonprimed mice (Fig. 4G). Depletion of CD4^+^ T cells abolished the enhancement of HEL-specific B cell proliferation, while the T cell-independent proliferation of HEL-specific B cells induced by HEL VLPs was maintained. The SW-HEL donor mice from which the HEL-specific B cells are derived are on the wild-type RAG background, and only up to 40% of the transferred CFSE-labeled B cells carry HEL^+^ BCR, while the rest of the B cells have undergone VH gene replacement (21) and did not bind HEL on their BCR (Fig. 4E). These HEL-negative B220^+^ CFSE^+^ B cells did not proliferate in any of the experimental groups (data not shown), confirming the antigen-specific nature of the B cell proliferation after VLP immunization. These results clearly demonstrate that the initial proliferation of naive B cells specifically recognizing VLP surface proteins was CD4^+^ T cell independent. However, Tetanol-induced CD4^+^ T cells were able to significantly increase this proliferation, indicating an effect of intrastructural help already on the early steps of antigen-mediated primary B cell expansion. To exclude the possibility that the proliferative response induced by the Tetanol immunization led to cell death, we also analyzed the effect of Tetanol priming on the expansion of HEL^+^ B cells in spleens on day 7 after HEL-VLP immunizations (Fig. 4H). Naive B cells from SW-HEL mice were transferred into Tetanol-primed and nonprimed mice which were then immunized with HEL-VLPs containing fragment C. As expected, priming with Tetanol led to a significant increase in the percentage of HEL^+^ B220^+^ B cells in the spleen (Fig. 4H), confirming their expansion.

### Improving the production of VLPs containing T helper cell epitopes of TT.

**FIG 5:**
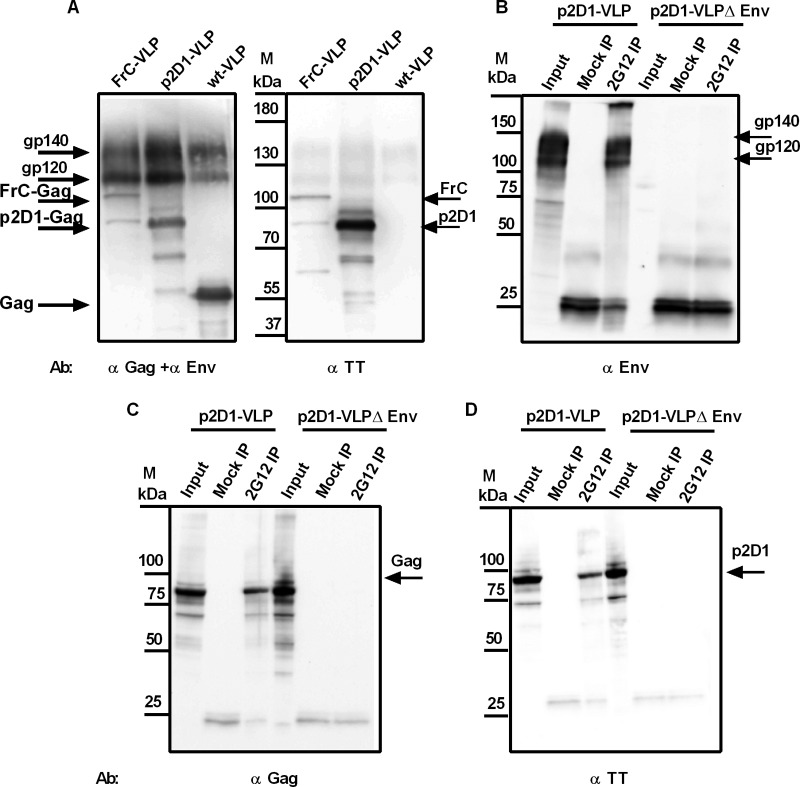
Characterization of p2D1-VLP. (A) Wild-type VLPs (wt-VLP), FrC-VLPs, and p2D1-VLPs were analyzed by Western blotting with an antiserum against gp120 of HIV Env (α Env), a monoclonal antibody against p24 of Gag (α Gag), and a monoclonal antibody against FrC and p2D1 (α TT). (B to D) p2D1-VLPs containing or lacking Env (p2D1-VLPΔEnv) were immunoprecipitated with 2G12 or mock treated (Mock IP). Precipitates were analyzed by Western blotting with the antibodies described for panel A. Equal amounts of p2D1-VLPs that were used for the immunoprecipitation were analyzed directly by SDS-PAGE (Input). The additional bands at 25 kDa in immunoprecipitation samples are due to protein G that was coeluted from the beads.

Since the yield of VLPs based on the FrC-Gag expression plasmid was low, we compared the efficiency of VLP formation by FrC-Gag with that by wild-type Gag and a novel Gag expression plasmid that only contained the p2 T helper cell epitope of fragment B and domain 1 of fragment C (Fig. 3A). Wild-type VLPs, FrC-VLPs, and p2D1-VLPs were prepared by cotransfection of the HIV Env expression plasmid with expression plasmids for wild-type Gag, FrC-Gag, or p2D1-Gag, respectively. As expected from the calculated molecular weight, the p2D1-Gag pelleted through a 35% sucrose cushion was detected by Western blotting with antibodies to Gag and fragment C of tetanus toxoid (TT) at 80 kDa (Fig. 5A). Although additional weaker bands, presumably due to proteolytic cleavage, could also be observed, the intensity of the p2D1-Gag band was substantially stronger than that of the FrC-Gag band. Consistent with more efficient particle formation, the Env content of p2D1-VLPs was also higher than the Env content of FrC-VLPs (Fig. 5A). To further confirm incorporation of HIV Env into the VLPs, the HIV Env expression plasmid was cotransfected with the p2D1-Gag expression plasmid. VLPs were purified from the supernatant of transfected cells and immunoprecipitated with the HIV Env antibody 2G12. Western blot analyses for HIV Env revealed the presence of HIV Env in the precipitates (Fig. 5B). Coprecipitation of Gag (Fig. 5C), which also reacted with an antiserum to fragment C (Fig. 5D), indicated that HIV Env, Gag, and fragment C were part of the same VLPs. The specificity of the immunoprecipitation was confirmed using VLPs lacking Env (Fig. 5B to D). Based on the more efficient particle formation, p2D1-Gag was selected for all subsequent experiments.

### Influence of the priming immunization on HIV Env-specific IgG subtype responses to VLP vaccines containing T helper cell epitopes of heterologous vaccine antigens.

**FIG 6:**
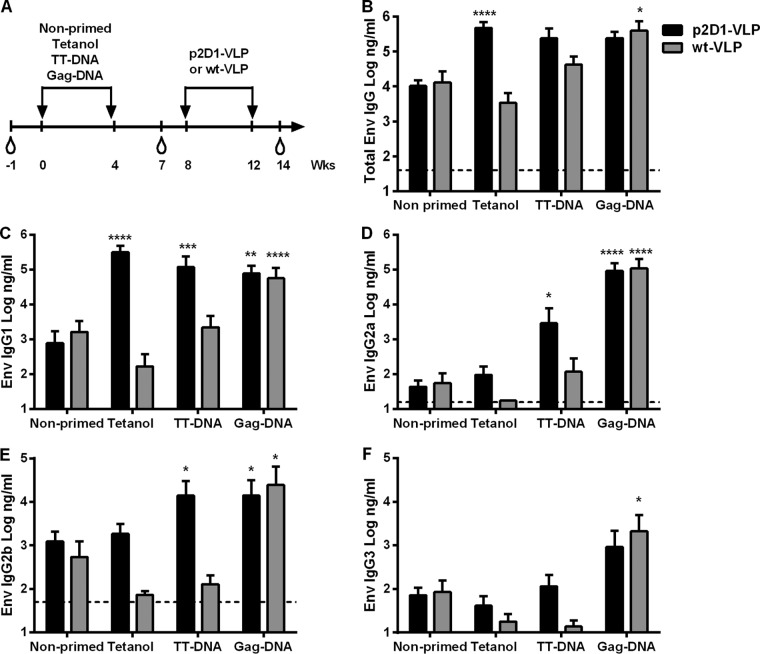
Requirements for the modulation of the Env-specific IgG subtype response by intrastructural help. (A) BALB/c mice were immunized at weeks 0 and 4 with Tetanol or DNA vaccines expressing tetanus toxoid or HIV Gag. All primed and nonprimed mice were boosted at weeks 8 and 12 with p2D1-VLPs or wild-type VLPs (wt-VLP). (B) Total Env-specific antibody concentrations in sera of mice primed with the indicated immunogens 2 weeks after the second immunization with the indicated VLPs. Shown are the mean values with SEM of logarithmically transformed HIV Env antibody concentrations in the sera of 12 to 18 animals per group from three independent experiments. *, *P* < 0.05; **, *P* < 0.01, ***, *P* < 0.001; ****, *P* < 0.0001 (Kruskal-Wallis test with Dunn's posttest; vaccine groups versus nonprimed). (C to F) Env specific IgG1 (C), IgG2a (D), IgG2b (E), and IgG3 (F) subtypes at 2 weeks after the second VLP immunization in animals boosted with p2D1-VLPs versus those boosted with wt-VLPs. Shown are the mean values with SEM of logarithmically transformed concentrations of Env-specific IgG subtypes in sera of 12 to 18 animals per group from three independent experiments. *, *P* < 0.05; **, *P* < 0.01, ***, *P* < 0.001; ****, *P* < 0.0001 (Kruskal-Wallis test with Dunn's posttest; vaccine groups versus nonprimed). (B, D, and E) The dashed lines represent the background values for naive sera. (C and F) The background for naive sera lies on *x* axis at a value of 1.

The results shown in Fig. 2F indicated that after priming with either adjuvanted Gag protein or Gag DNA, intrastructural help increased the IgG2a/IgG1 ratio of anti-HIV Env antibodies after the VLP immunization. In contrast, priming with Tetanol enhanced only the HIV Env IgG1 antibody responses to FrC-VLP immunizations (Fig. 3C and D). To further confirm that the HIV Env-specific IgG subtype response was dependent on the type of T helper cells mediating intrastructural help, we compared the effects of Tetanol priming on the HIV Env antibody responses to VLPs containing (p2D1-VLPs) or lacking (wt-VLPs) the p2D1 fragment. To reveal whether the formulation and/or the nature of the antigen can modulate the IgG subtype response, we also included tetanus toxoid and Gag DNA vaccines for priming (Fig. 6A).

Priming with Tetanol or TT DNA enhanced HIV Env-specific antibody levels in response to p2D1-VLPs only, while the Gag DNA priming immunizations modulated HIV Env antibody responses to both types of VLP vaccines to similar extents (Fig. 6B to F). Interestingly, the HIV Env IgG subtype response to the same p2D1-VLP booster immunizations clearly differed for the different priming immunizations (Fig. 6C to F). Priming with Tetanol significantly enhanced only HIV Env IgG1 antibody levels, while priming with TT DNA enhanced IgG1, IgG2a, and IgG2b antibody levels. Priming with Gag DNA led to an approximately 2,000-fold-higher mean Env-specific IgG2a antibody levels in comparison to those in the nonprimed groups (Fig. 6D), while HIV Env IgG1 antibody levels increased 50- to 100-fold (Fig. 6C).

**FIG 7:**
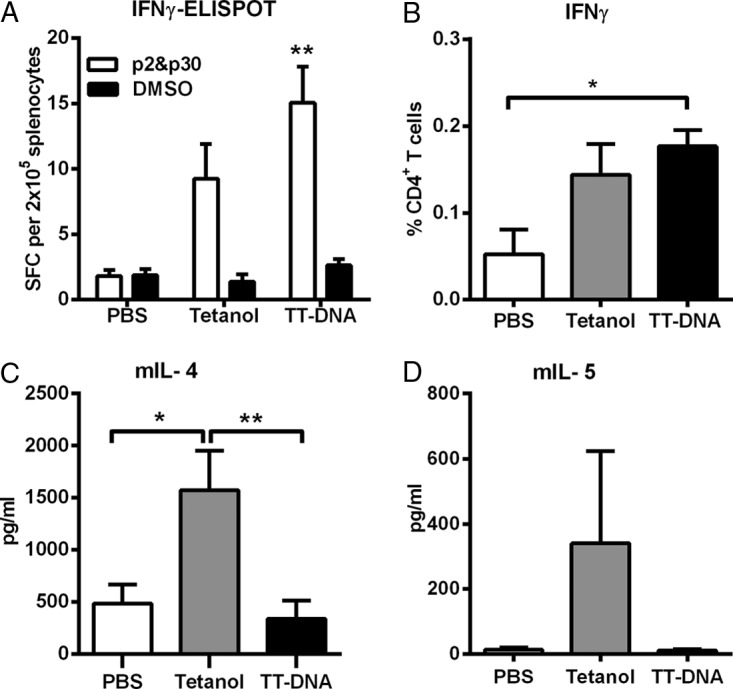
Characterization of tetanus toxoid-specific cellular immune responses. BALB/c mice were immunized i.m. once with PBS, Tetanol, or TT DNA. (A) TT-specific T cell responses 2 weeks after immunization as determined by an IFN-γ ELISPOT assay with splenocytes stimulated with MHC-II-restricted p2 and p30 T helper peptides derived from TT. Shown are the mean values with SEM of spot-forming cells (SFC) producing IFN-γ per 200,000 splenocytes for 8 animals per group out of two independent experiments. **, *P* < 0.01 (one-way ANOVA with Tukey's posttest; vaccine groups versus PBS). (B) Percentage of splenic CD4^+^ T cells cell producing IFN-γ after in *vitro* stimulation with p2 and p30 T helper peptides as measured by intracellular cytokine staining. Mice (6 per group) were sacrificed 2 weeks after immunization. The cells were stained for surface expression of CD4 and for intracellular expression of IFN-γ. The background values for unstimulated cultures were subtracted. Shown are the mean values with SEM. *, *P* < 0.05 (one-way ANOVA with Tukey's posttest; vaccine groups versus PBS). (C and D) Tetanus-specific IL-4 (C) and IL-5 (D) cytokine secretion as determined by ELISA. Two weeks after immunization, splenocytes were stimulated with p2 and p30 T helper peptides for 48 h. The mean values with SEM for 10 animals out of two independent experiments are shown. *, *P* < 0.05 (Tetanol versus PBS); **, *P* < 0.01 (Tetanol versus TT DNA).

To correlate the differential IgG subtype responses to differences in T helper cells mediating intrastructural help, TT-specific CD4^+^ T cell responses were characterized after the priming immunization. Enzyme-linked immunosorbent spot (ELISPOT) analyses and intracellular cytokine staining (ICS) after stimulation with the MHC-II-restricted peptides p30 and p2 revealed induction of T helper cells producing IFN-γ (Fig. 7A and B), which showed a trend of being more pronounced in the TT DNA vaccine group. For other Th1 cytokines (TNF-α and interleukin-2 [IL-2]), no difference between the two groups was observed (data not shown). In contrast, secretion of the Th2 cytokines IL-4 and IL-5, could be detected only in Tetanol-immunized animals (Fig. 7C and D).

### Influence of Tetanol priming on the quality of the HIV Env antibody response to VLP vaccines containing TT T helper cell epitopes.

**FIG 8:**
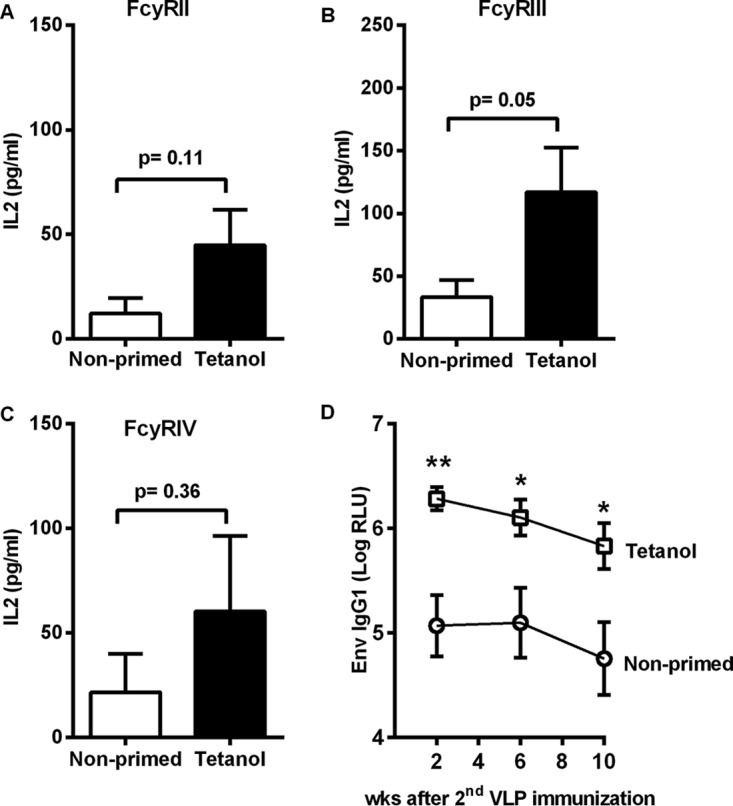
Fcγ receptor activation assays and durability of the humoral immune response. BALB/c mice (6 per group) were immunized twice at weeks 0 and 4 with Tetanol. Primed and nonprimed mice were boosted at weeks 8 and 12 with p2D1-VLPs. (A to C) FcγR activation by Env-specific Abs from sera taken 2 weeks after the second VLP immunization using FcγRII (A), FcγRIII (B), or FcγRIV (C) reporter cell lines secreting IL-2. Background values from parental P815 cells not expressing Env were subtracted from the values generated with P815Env cells to obtain the Env-specific Fcγ receptor activation. Shown are the mean IL-2 concentrations with SEM for 6 immune sera per group at a 1:100 dilution. *, *P* < 0.05 (Tetanol-primed versus nonprimed; unpaired *t* test). (D) Env IgG1 antibody levels were monitored for 10 weeks after the second VLP immunization. Shown are the mean values with SEM of logarithmically transformed RLUs in the sera of 6 animals per group (*, *P* < 0.05; **, *P* < 0.01, Tetanol-primed versus nonprimed, one-way ANOVA with Tukey's posttest).

To further characterize the effect of Tetanol priming on the quality of the antibody response, we analyzed Fc-effector functions of HIV Env-specific antibodies 4 weeks after the second p2D1-VLP booster immunization by reporter cell-based activation assays for Fc-γRII, Fc-γRIII, and Fc-γRIV (9). Sera from Tetanol-primed and p2D1-VLP-boosted mice led to a stronger activation of all three Fc-γ receptors than with sera from mice only receiving the p2D1-VLP immunizations, although these differences did not reach statistical significance (Fig. 8A to C).

To exclude that intrastructural help only transiently enhances antibody responses, a 10-week serological follow-up was also performed. Clearly, HIV Env-specific IgG1 antibody levels induced by p2D1-VLP immunization continued to be significantly higher in Tetanol-primed animals than in nonprimed controls (Fig. 8D).

## DISCUSSION

Similar to the case with gene-based vaccines encoding Gag (8, 9), the adjuvanted Gag protein vaccine also induced T helper cells at levels sufficient to provide intrastructural help. However, for the protein vaccine this clearly required a strong adjuvant, such as poly(ICLC). Extending the concept of intrastructural help from virus-specific T helper cells, the results also constitute proof of concept for the strategy that T helper cells induced by licensed vaccines can be harnessed to provide help for B cell responses induced by VLP vaccines. The observation that the enhancement of antibody responses to HIV Env by prior immunization with tetanus vaccines is entirely dependent on the incorporation of tetanus toxoid-derived protein fragments into the VLP indicates that it is not a general modification of the inflammatory milieu during priming that persists and mediates the modulation of the Env antibody response at the time of the VLP immunization. Consistently, depletion of CD4^+^ T cells abolished the enhancement of the proliferation of BCR-transgenic B cells specific for the model surface antigen of the VLP by the Tetanol priming immunization.

Interestingly, both the antigen and the type of the priming immunization affected the IgG subtype responses to HIV Env after the VLP immunization (Fig. 2 and 6). Tetanol priming strongly enhanced the IgG1 Env antibody response and had no effect on the anti-Env IgG2a, IgG2b, and IgG3 responses. Priming with a DNA vaccine encoding tetanus toxoid fragment C led to a strong increase in IgG1 and a modest increase in IgG2a and IgG2b responses to HIV Env. These IgG subclass distributions may reflect the Th1/Th2 cytokine profile of the antigen-specific CD4^+^ T cells induced by priming. Priming with Tetanol raised TT-specific CD4^+^ T cells with a prominent Th2 phenotype. Intrastructural help by these T helper cells during the VLP booster immunizations may trigger expansion of B cells secreting Env-specific IgG1 and block class switching to other IgG subtypes. On the contrary, TT DNA immunization induced tetanus-specific CD4^+^ T cells with a Th1-dominated phenotype most likely imprinting the Th1 phenotype to Env-specific B cells during the VLP booster immunizations (Fig. 7). The difference in the IgG subtype responses may directly affect Fc effector functions of the HIV Env-specific antibodies, as observed previously (9). In the current study, intrastructural help also enhanced the Fc effector function of the immune sera (Fig. 8A to C). Whether this is due to higher overall level of HIV-1 Env-specific antibodies, changes in the ratio of IgG subtypes, or differences in the glycosylation of the antibodies, remains to be determined. Intrastructural help provided by Tetanol priming also resulted in a long-lasting increase of the IgG1-dominated humoral immune response (Fig. 8D), suggesting formation of long-lived plasma cells.

The applicability of intrastructural help in humans will critically depend on the efficient incorporation of protein fragments of licensed vaccines into the VLPs. As shown by the inefficient particle formation by FrC-Gag, the size of the fragment that can be incorporated and/or its folding may be limiting. Therefore, it may be advantageous to incorporate linear T helper cell epitopes rather than entire protein domains. However, the allelic variation of MHC-II in the human population may require the identification of MHC-II epitopes to which many individuals raise T helper cell responses or the incorporation of a panel of T helper cell epitopes. The use of promiscuous or universal T helper epitopes that are binding on a broad range of MHC-II human alleles might be an elegant solution to overcome this problem. The tetanus toxoid sequence even offers two of such epitopes: p2 and p30 (22). Although each of the epitopes demonstrates MHC-II binding on multiple MHC-II alleles among different species (from mouse to human), helper T cell activity might strongly vary between the alleles (23–25). Our observation that VLPs containing FrC with only one epitope (p30) were more potent in BL/6 (H-2^b^) than in BALB/c (H-2^d^) mice (Fig. 3) is consistent with previous reports (23). Reduction of total FrC size with additional incorporation of the second universal epitope (p2) led to efficient particle formation and improved humoral immune responses in BALB/c mice (Fig. 6). Since each HIV particle contains approximately 2,000 Gag molecules, we assume a similar number of molecules of the T helper cell epitopes. However, the precise number of epitope molecules required for efficient intrastructural help remains to be determined. The merit of incorporating protein fragments derived from the tetanus toxoid is that most people have been previously vaccinated with Tetanol and therefore are likely to have preexisting memory T cells. Follow-up studies will explore whether preexisting memory T cells can be directly recruited by intrastructural help or whether Tetanol booster immunizations are required prior to the VLP immunizations. However, given the fact that the type and persistence of antibody responses are thought to be largely regulated by T helper cells, the intrastructural help approach seems to offer plenty of opportunities to improve the antibody response to VLP vaccines.

## MATERIALS AND METHODS

### Cell lines, plasmids, and VLP preparation.

HEK293T cells were cultured in Dulbecco's modified Eagle medium (DMEM) (Life Technologies, Darmstadt, Germany) with 10% heat-inactivated fetal calf serum (FCS) (Life Technologies, Darmstadt, Germany) and 250 μg/ml gentamicin (Applichem, Darmstadt, Germany). Gag and p2D1-Gag expression plasmids were constructed by inserting the coding sequence for fragment C (FrC) (amino acids [aa] 865 to 1316 of tetanus toxoid [TT] according to GenBank entry number AAA23282.1) (23, 26, 27) or for the p2D1 fragment spanning the promiscuous Th epitope p2 (QYIKANSKFIGITE) and domain 1 of FrC (FrCD1) (aa 865 to 1120 according to GenBank entry number AAA23282.1) by overlap extension PCR between the 3′ codons (DTGHSSQ) of matrix (MA) and the 5′ codons (VSQNYPI) of capsid (CA) of the HgSyn expression plasmid (28). The plasmids HgSyn (a codon-optimized HIV Gag expression plasmid) (28), pConBgp140GCD (encoding codon-optimized HIV-Env clade B consensus sequence aa 1 to 703 of GenBank entry number ABG67916.1 fused to the intracellular domain of vesicular stomatitis virus G [VSV-G] [aa 97 to 122 of GenBank entry number: CAA24524.1]) (8, 29), and pC-HEL-TM (encoding the membrane-anchored form of HEL) (20) have been described previously. All plasmids were prepared with the NucleoBond Xtra Maxi EF or the PC10000 EF kit (Macherey-Nagel, Dueren, Germany). VLPs were prepared as described previously with slight modifications (8, 30). Briefly, 293T cells were cotransfected with 30 μg of one of the Gag expression plasmids HgSyn, FrC-Gag, or p2D1-Gag and 30 μg pConBgp140GC/D to generate wild-type VLPs, FrC-VLPs, and p2D1-VLPs, respectively. HEL-VLPs containing HEL on their surfaces and FrC-Gag inside were produced by cotransfection of 30 μg of FrC-Gag and 30 μg of pC-HEL-TM. Transfections were performed in 175-cm^2^ flasks (Greiner Bio One, Frickenhausen, Germany) with 1 μg polyethylenimine per 1 μg DNA. At 6 h posttransfection, the medium was replaced with fresh DMEM free of FCS and containing only 1% gentamicin (Applichem) and 1% GlutaMAX (Life Technologies). Two days later, VLPs were purified from the supernatant of transfected cells by ultracentrifugation through a 35% sucrose cushion for 4 h at 90,000 × *g* and 4°C. Finally, the purified VLPs were resuspended in sterile phosphate-buffered saline (PBS) and stored at 80°C until further use. To determine the amount of Env and Gag in the VLP preparations, high-binding microtiter plates (Greiner Bio One) were coated with the purified VLPs or serial dilutions of known amounts of p55 Gag (31) or HIV gp120 (HIV-1 IIIB; NIH Reference and Reagent Program) as standards. The amounts of p55 and HIV gp120 from the VLP preparations bound to the microtiter plate were then determined using an anti-HIV-1 p24 monoclonal antibody (183-H12-5C; NIH AIDS Reference and Reagent Program), the monoclonal gp120 antibody 2G12 (Polymun, Klosterneuberg, Austria), and matched horseradish peroxidase (HRP)-conjugated secondary antibody reagents. The endotoxin levels of the immunogens were determined with the Limulus amebocyte lysate assay (QCL-1000; Cambrex, Walkersville, MD) to be below 0.1 unit per injection dose.

### Immune precipitation of p2D1-VLPs.

To show that the p2D1-VLPs incorporate Env, Gag, and the p2D1 fragment of TT into the same particles, we performed an immune precipitation with p2D1-VLPs as described before (9). To this end, we coated 50 μl protein G Dynabeads (Invitrogen, Life Technologies) with 12 μg monoclonal human anti-Env Ab 2G12 (Polymun, Klosterneuburg, Austria) in PBS plus 0.05% Tween 20 (PBS-T) for 30 min at room temperature. After removal of excess 2G12 and washing steps with PBS-T and PBS, we incubated the Dynabeads with p2D1-VLPs containing or lacking Env for 30 min at room temperature. Following extensive washing with PBS, bound VLPs were eluted with reducing SDS sample buffer by boiling for 5 min at 70°C and analyzed by Western blotting. HIV-1 Env was detected with a polyclonal goat anti-gp120 Ab (Acris, Herford, Germany), Gag proteins with a monoclonal murine anti-p24 Ab (183-H12-5C; National Institutes of Health AIDS Reagent Program), and p2D1 and FrC with a polyclonal antiserum against TT (POL 016 anti-tetanus toxin; Statens Serum Institute, Copenhagen, Denmark) (32), with HRP-conjugated secondary antibodies P0449, P0447, and P0399 (DakoCytomation, Glostrup, Denmark), respectively.

### Mice and immunizations.

BALB/cJRj (BALB/c) and C57BL/6J (BL/6) mice (6 to 8 weeks old) were purchased from Janvier Laboratories (Saint-Berthevin, France), and 12-week-old SW-HEL mice from in-house breeding were donors for HEL-specific B cells for adoptive transfer experiments. All mice were housed in individual ventilated cages in accordance with the national law and institutional guidelines at the animal facility of the Faculty of Medicine, Ruhr University Bochum (Bochum, Germany). For the DNA immunization, the animals were anesthetized by intraperitoneal (i.p.) injection of 100 mg/kg body weight ketamine (CP-Pharma, Burgdorf, Germany) and 15 mg/kg body weight xylazine (CP-Pharma). The TriGrid electrode array (Ichor Medical, San Diego, CA) with 2.5-mm electrode spacing bearing the centered injection needle was inserted into the shaved hind legs of the mice (23). A volume of 50 μl PBS containing 30 μg plasmid DNA was injected intramuscularly (i.m.) into the gastrocnemius muscle of each hind leg, which was immediately followed by the local application of electrical signals of 63-V amplitude and 40-ms total duration. The VLPs were diluted in sterile PBS. All animals received 400 ng of Env per immunization in a total volume of 100 μl by subcutaneous (s.c.) injection distributed to both hind footpads. A volume of 50 μl PBS containing 1 μg of Gag protein (p55) (31) alone or in combination with 0.3, 1, 3, or 10 μg poly(ICLC) (Hiltonol-Oncovir, Inc.) was injected i.m. into each hind leg. For Tetanol immunization, mice received a volume of 100 μl PBS containing 10 μl Tetanolpur (Novartis Vaccines and Diagnostics, Marburg, Germany) by i.m. injection distributed to both hind legs.

### Characterization of cellular immune responses.

HIV Gag-specific CD4^+^ T cell responses and tetanus-specific CD4^+^ T cell responses in spleens from mice were determined by intracellular cytokine staining (ICS), cytokine-specific enzyme-linked immunosorbent assay (ELISA) as described before (9), and IFN-γ ELISPOT. To this end, mice were sacrificed 2 weeks after a single immunization, the spleens were removed, and single-cell suspensions were prepared using a 70-μm cell strainer (BD Biosciences, Heidelberg, Germany). After red blood cell (RBC) lysis, splenocytes were resuspended at 10^7^ cells/ml in RPMI 1640 supplemented with 10% heat-inactivated FCS, 1% penicillin-streptomycin, 10 μmol HEPES, and 4 μmol l-glutamine (all from Life Technologies, Thermo Fisher Scientific) and 50 μmol β-mercaptoethanol. For ICS, 10^6^ splenocytes/well were seeded in a 96-well U-bottom microtiter plate (Nunc; Thermo Fisher Scientific) and stimulated with 5 μg/ml MHC-II-restricted peptides PVGEIYKRWIIL and SPEVIPMFSALSEGA for HIV-1 Gag or 2 μg/ml MHC-II-restricted peptides p2 (QYIKANSKFIGITELK) and p30 (FNNFTVSFWLRVPKVSASHLE) for tetanus toxoid in the presence of 2 μg/ml anti-CD28 (37.51; Life Technologies, Thermo Fisher Scientific) and 2 μmol monensin for 6 h at 37°C in a humidified 5% CO_2_atmosphere. After stimulation, the cells were surface stained with anti-mouse CD4 peridinin chlorophyll protein (PerCP)–eFluor710 (RM4-5) and fixable viability dye eFluor780 (both from Life Technologies, Thermo Fisher Scientific). Following fixation with 2% paraformaldehyde, cells were permeabilized with 0.5% saponin in the presence of 1.7 μg/ml anti-mouse CD16/CD32 (93; Life Technologies, Thermo Fisher Scientific) and subsequently stained with anti-mouse TNF-α–phycoerythrin (PE)-Cy7 (MP6-XT22) and anti-mouse IFN-γ–PE (XMG1.2) (both from Life Technologies, Thermo Fisher Scientific) and anti-mouse IL-2–allophycocyanin (APC) (JES6-5H4; Life Technologies, Thermo Fisher Scientific). Data were acquired on an FACSCanto II (BD Biosciences) and analyzed with FlowJo (Tree Star, Ashland, OR, USA). The gating strategy was reported previously (9). For the cytokine-specific ELISA, 5 × 10^6^ cells/well were seeded into 48-well plates and stimulated with 2 μg/ml MHC-II-restricted peptides of tetanus toxoid (p2 and p30) in the presence of 2 μg/ml anti-CD28 (37.51; Life Technologies, Thermo Fisher Scientific) for 48 h at 37°C in a humidified 5% CO_2_ atmosphere. After the stimulation, the supernatants were analyzed for the presence of IL-4 and IL-5 with the cytokine-specific Ready-SET-Go ELISA (Life Technologies, Thermo Fisher Scientific) according to the manufacturer's protocol. For ELISPOT assay, IFN-γ responses were detected with a mouse IFN-γ ELISPOT kit (88-7384; Life Technologies, Thermo Fisher Scientific). Briefly, 96-well Multiscreen-IP filter plates (Millipore) were pretreated with 20 μl of 70% methanol for 30 to 60 s and then washed three times in PBS (Ca^2+^/Mg^2+^ free), followed by coating with 100 μl of capture antibody. The plates were incubated overnight at 4°C. Excess coating antibody was removed, and plates were washed 2 times with 200 μl of ELISA/ELISPOT coating buffer (Life Technologies, Thermo Fisher Scientific) and then were blocked in complete RPMI 1640 medium for 1 h at 37°C before cells were added. Medium was removed from the plates, splenocytes in single-cell suspension were added at 2 × 10^5^ cells in duplicates to the wells, and then the cells were stimulated by tetanus p2 and p30 peptides at a final concentration of 2 μg/ml. Wells containing complete RPMI 1640 medium and 10% dimethyl sulfoxide (DMSO) (as in peptide-stimulated cultures) served as negative controls. Plates were incubated at 37°C for 72 h and then washed 3 times with ELISA/ELISPOT washing buffer. Subsequently, 100 μl of biotinylated detection antibody was added to each well. Plates were then incubated at room temperature for 2 h before washing 4 times with ELISA/ELISPOT washing buffer. Next, 100 μl Avidin-HRP was added per well and incubated at room temperature for 45 min. After washing the plates 3 times with ELISA/ELISPOT washing buffer, 100 μl of freshly prepared 3-amino-9-ethylcarbazole (AEC) substrate was added to each well. Plates were left at room temperature until spots developed (approximately 3 to 5 min) and then washed with water to stop the reaction. Plates were allowed to dry and then read in an ELISPOT plate reader (Immunospot; CTL Europe GmbH, Bonn, Germany). Spots were counted using AID software (Autoimmune Diagnostika, Germany). For analysis, the number of background spots (DMSO treated) was subtracted from the number of spots detected in wells containing the p2 and p30 peptides.

### CD4^+^ T cell depletion and *in vivo* B cell proliferation and expansion assays.

Tetanus-specific CD4^+^ T cells were depleted by 3 injections of purified anti-mouse CD4 antibody (GK1.5; BioLegend, Germany). As described before (20), SW-HEL B-cells (unlabeled or labeled with CFSE) were transferred to recipient BL/6 mice (3 × 10^6^ to 5 × 10^6^ cells per mouse) by intravenous (i.v.) injection. On the same day, recipient mice were immunized with FrC-VLP-HEL containing FrC by i.v. injection at a final dose of 100 ng HEL per mouse. After 72 h, splenocytes were isolated and stained with HEL-Alexa 647 and B220 APC-eFluor780. The CFSE fluorescence intensity of CFSE^+^ HEL^+^ B220^+^ cells was determined by flow cytometry.

### Analyses of humoral immune responses.

Mice were bled by puncture of the retroorbital sinus with a heparinized 10-μl hematocrit capillary (Hirschmann Laborgerate, Eberstadt, Germany). The sera were obtained after 5 min of centrifugation at 2,600 × *g* in a tabletop centrifuge and stored at −20°C until further use. Antibody responses against HIV-1 Gag and HIV-1 Env were determined by Ag-specific ELISA as previously described (8). Briefly, 96-well high-binding microtiter plates (Greiner Bio-One) were coated with 200 ng glutathione *S*-transferase (GST)–Gag or 200 ng ConB gp120 in 0.1 mol bicarbonate buffer (pH 9.6) per well at room temperature overnight. After washing with PBS-T, the wells were blocked with 5% skim milk powder in PBS-T and washed again before they were incubated with the various sera at different dilutions in blocking buffer. After washing, bound antibodies were incubated with HRP-conjugated anti-mouse antibodies (P0447; DakoCytomation, Glostrup, Denmark) and washed extensively. Bound HRP-conjugated antibodies were detected with an ECL solution composed of 5 ml Luminol solution (3-aminophtolhydrazide; Sigma-Aldrich 09253-25G), 50 μl solution B (*p*-coumaric acid; Sigma-Aldrich C9008-5G), and 1.6 μl 30% H_2_O_2_ (Merck KGaA, Darmstadt, Germany) in an Orion-96 microplate reader (Berthold, Bad Wildbad, Germany). Humoral immune responses for Gag and tetanus toxoid are expressed as log_10_-transformed relative light units (RLU). To determine Env-specific IgG1, IgG2a, IgG2b, and IgG3 antibody concentrations, recombinant b12 antibodies containing the different Fc regions were expressed in 293T cells and purified with protein G-Sepharose 4 Fast Flow instrument (GE Healthcare Amersham, Darmstadt, Germany). After quantification of the concentrations of the different b12 IgG subtypes by a sandwich ELISA with AffiniPure goat anti-mouse IgG (Jackson ImmunoResearch, USA) as capture antibody and HRP-labeled, polyclonal rabbit anti-mouse sera recognizing IgG1, IgG2a, IgG2b, and IgG3 (5300-01B; SouthernBiotech, Birmingham, USA) as detecting antibodies, they were used as standards in the IgG subtype-specific Env ELISAs. Bound IgG1, IgG2a, IgG2b, or IgG3 antibodies were detected, after a washing step, with HRP-conjugated Abs specific for each of the IgG subtypes (X56 and R19-15 from BD Biosciences, Heidelberg, Germany, and SB74g and SB76b from Southern Biotech, Birmingham, AL, USA).

### FcγR activation assay.

Activation of each murine Fcγ receptor (FcγR) by the different immune sera was analyzed as described previously (9). Briefly, parental P815 cells and P815 Env cells expressing ConB-gp140GC/D were incubated with 1:100 dilutions of the different sera in 100 μl PBS for 30 min at room temperature in a 96-well V-bottom plate. After a washing step with PBS, either mFcγRII-CD3ζ-, mFcγRIII-CD3ζ-, or mFcγRIV-CD3ζ-transduced BW5147 reporter cells (9) were added in 100 μl culture medium and mixed, and the cocultures were incubated at 37°C in a humidified 5% CO_2_ atmosphere. Eighteen hours later, 100 μl PBS supplemented with 10% FCS and 0.1% Tween 20 was added to each well and completely mixed to favor the release of murine IL-2 from the BW5147 cells. After 15 min of incubation at room temperature, the cells were pelleted, and the IL-2 concentrations in the supernatants were determined by ELISA with purified capture and biotinylated detection antibodies (BD Biosciences), as previously described (9).

### Ethics statement.

All animal experiments performed during this study were approved by an external ethics committee authorized by the North Rhine-Westphalia Ministry for Environment and Nature Protection, Agriculture and Consumer Protection (licenses 84-02.2011.A111, 84-02.04.2012.A220 and 84-02.04.2013.A052). All mice were handled according to the instructions of the Federation of European Laboratory Animal Science Association.

### Statistical analysis.

Quantitative summary data are shown as means ± standard errors of the means (SEM). Statistical evaluation was performed with GraphPad Prism software version 6 using unpaired *t* tests, one-way analysis of variance (ANOVA) with Tukey's posttest, or the Kruskal-Wallis test with Dunn's posttest, as indicated in the figure legends.
