# Correction for Elsayed et al., “Intrastructural Help: Harnessing T Helper Cells Induced by Licensed Vaccines for Improvement of HIV Env Antibody Responses to Virus-Like Particle Vaccines”

**DOI:** 10.1128/jvi.01954-21

**Published:** 2022-02-09

**Authors:** Hassan Elsayed, Ghulam Nabi, William J. McKinstry, Keith K. Khoo, Johnson Mak, Andres M. Salazar, Matthias Tenbusch, Vladimir Temchura, Klaus Überla

**Affiliations:** a Institute of Clinical and Molecular Virology, University Hospital Erlangen, Friedrich-Alexander Universität Erlangen-Nürnberg, Erlangen, Germany; b Department of Microbial Biotechnology, Genetic Engineering and Biotechnology Division, National Research Centre, Dokki, Giza, Egypt; c Department of Molecular and Medical Virology, Ruhr-University Bochum, Bochum, Germany; d CSIRO Manufacturing, Parkville, Victoria, Australia; e School of Medicine, Deakin University, Geelong, Australia; f Institute for Glycomics, Griffith University, Gold Coast, Queensland, Australia; g Oncovir, Inc., Washington, DC, USA

## AUTHOR CORRECTION

Volume 92, no.14, e00141-18, 2018, https://doi.org/10.1128/JVI.00141-18. Page 14: The first sentence of Acknowledgments should be replaced with the following.

This project has received funding from the European Union’s Horizon 2020 research and innovation programme under grant agreement no. 681137, from the Deutsche Forschungsgemeinschaft (transregional collaborative research grant TRR60), and through the Collaboration on AIDS Vaccine Discovery (CAVD) by the Bill & Melinda Gates Foundation.

